# Thermomechanics analysis of cryopreservation by vitrification of the popliteal artery

**DOI:** 10.1371/journal.pone.0350078

**Published:** 2026-08-18

**Authors:** Devarsh M. Vispute, Yoed Rabin

**Affiliations:** Department of Mechanical Engineering, Biothermal Technology Laboratory, Carnegie Mellon University, Pittsburgh, Pennsylvania, United States of America; Cardiff Metropolitan University, UNITED KINGDOM OF GREAT BRITAIN AND NORTHERN IRELAND

## Abstract

This study aims at exploring packaging configurations and thermal protocols to reduce thermomechanical stress during cryopreservation by vitrification of the popliteal artery and thereby reducing the risk of structural damage. This study explores the potential advantages of preserving the blood vessel in spiral and helical configurations, in solid and hollow cylindrical containers. This study includes ideal rewarming scenarios of convective rewarming, nanowarming, and a combination of both. Results of this study indicate that the maximum stress in the blood vessel is comparable in all configurations examined and may be close to structurally hazardous levels. The maximum stress in the surrounding cryoprotective medium, however, might rise to much higher levels, exceeding the strength to fracture of the material. Once a fracture is initiated somewhere in the container it can rapidly propagate across it, and even through areas where the average stresses are lower. This study demonstrates that thermomechanical stress can be lowered below hazardous levels by careful selection of nanoparticle concentrations, in combination with a matching convective boundary condition. This study demonstrates how this combination can be tailored to various configurations. Finally, a special case is considered to explore the effects of transferring the specimen between cooling instrumentation during cryogenic storage. This study demonstrates that even a brief exposure to ambient temperature of 1 min or less can cause sudden stress spikes in the specimen, potentially exceeding the strength of the material. This special case demonstrates the need to carefully consider the feasibility and practicality of lab protocols when aiming to reduce the likelihood of structural damage.

## 1. Introduction

Peripheral artery disease (PAD) poses a serious public health issue worldwide [1]. As the third common manifestation of atherosclerosis after coronary artery disease and stroke, PAD reduces the blood supply to the limbs which can lead to ischemia [1]. More than 113 million people lived with PAD globally in 2019, when more than 10 million new cases and 74,063 consequential deaths were reported worldwide [1]. It is estimated that more than 8.5 million adults are affected by PAD in USA, with 11,753 deaths reported in 2019 with PAD as the underlying cause [2]. In addition to direct economic burden on the public health system, PAD results in substantial additional costs through reduced productivity [1].

Depending on the severity of symptoms, the location of the artery and its length, PAD can be treated through endovascular intervention or bypass surgery [3]. The popliteal artery is uniquely affected by forces applied by body movements due to its location, resulting in bending, torsion, and compression. In turn, these forces increase the complexity of endovascular treatment for popliteal artery when compared with other sections of peripheral arteries [3]. While endovascular treatments can be effective for smaller lesion lengths, lower extremity bypass is considered to have better patency for longer lesions [3]. For optimal results in bypasses, it is recommended to use a good quality greater saphenous vein, or a prosthetic graft [3]. However, the patency of the graft reduces as the time progresses, requiring graft revision within one to five years of initial bypass surgery in many cases [3]. Since PAD patients often have accompanying cardiovascular disease [1], a suitable autogenous vein graft may not be available for initial bypass or revision surgeries in more than 20% of the cases [3]. As a potential alternative for autologous vascular grafts, tissue engineered blood vessels (TEBV) have shown promising results without functional limitations over long durations [4]. However, the high manufacturing time and cost makes TEBV unsuitable for patients requiring frequent surgical interventions [4]. If a suitable vein graft, either autogenous or TEBV, can be made available on demand, the graft patency and limb-salvage rates can be improved – a case for which cryopreservation of blood vessel can serve as an enabling means. In concept, a bank of cryopreserved popliteal artery segments can be developed for autologous saphenous veins and manufactured-in-advance TEBVs, which will help reduce the wait time for initial and revision graft surgeries.

Despite successful cryopreservation of small-size specimens as reported in previous studies [5–7] and recent progress in vitrification of small organs [8], scaling up to large-size specimens, such as complex tissues and whole organs is associated with additional challenges to cryopreservation success [9–14]. Vitrification (*Vitreous* in Latin means *glassy*) is a promising alternative for long-term storage in cryogenic temperatures, where the biological material is stored in an ice-free state [15,16]. Ice suppression during vitrification is facilitated by rapidly cooling a specimen loaded with highly viscous cryoprotective agents (CPAs). The temperature gradients across the specimen increase with its size, which may give rise to thermomechanical stresses (interchangeable with *thermal stress* in this study) [15–19]. Specifically, thermal stress in the specimen develops due to differential thermal expansion, which may be caused by those temperature gradients [18,20]. Additional contributors to thermal expansion and thereby thermal stress are phase change effects [21], difference in thermal expansion within the specimen and its surroundings [14,22], and thermal expansion mismatch between specimen and container walls [13,19]. While thermal stress during cooling is a commonly expected effect, it has been shown that the thermal stress at the onset of rewarming only intensifies as the specimen is recovered from cryogenic storage, making it even more susceptible to structural damage [17,23,24]. Using computation tools, the current study assesses the feasibility of cryopreserving the popliteal artery by vitrification. More specifically, this study focuses on the ability to reduce the likelihood to structural damage due to thermal stress by designing better packaging configuration for the artery, as well by utilizing volumetric heating applications such as nanoparticles rewarming (also known as *nanowarming*) [23,25,26].

The preservation of blood vessels has been studied previously in order to meet the shortage of good quality allografts for bypass surgeries [27–29], and means to prevent manifestation of structural damage in the form of fracturing have been proposed [30,31]. Various experimental and modeling investigations have been conducted to study loading of CPA in the blood vessel [25,32–35], vitrification and crystallization effects [14,30,33,36], fracture formation [30,31,36–38], and nanowarming [25,32,39]. Cryomacroscopy was used to visualize thermo-physical events occurring along the vitrification process of carotid artery segments of 25 mm length [36,38], and to correlate functional recovery of arteries when marginal thermal conditions are applied [37]. Measurements of the stress-strain relationship and thermal expansion coefficient of blood vessels have been reported for vessel segments of 30 mm in length [40–44]. To reduce the likelihood of crystallization and the level of thermal stress during the rewarming portion of the cryoprotocol, nanoparticles and thin metal forms have been experimented with carotid arteries segments of 10 mm in length and varying thicknesses [25,32,39]. Vitrification and recovery of blood vessels have been investigated experimentally and computationally to analyze limiting cases of partial vitrification [14,22]. To the best of our knowledge, no thermal stress study has been conducted for vitrification of full-size popliteal arteries under realistic vitrification conditions.

Successful vitrification of popliteal arteries requires optimization of numerous parameters including the selection of CPA, thermal protocol, rewarming method, and storage containers. For large organs such as the heart, kidney and liver, the selection of container geometry is limited by shape and size of the organ. A popliteal artery, on the other hand, resembles a long flexible tube which can be packed in various shapes and container volumes. By relaxing the constraints on the container’s geometry, it is feasible to select configurations more favorable to reduce the adverse effects of thermal stress and help preserve the structural integrity of the blood vessel. Using thermomechanics stress analysis, the current study assesses the potential advantages of two basic configurations: spiral and helical arrangement of a blood vessel (BV) in a cylindrical container. Note that this study focuses on reducing thermal stress in the blood vessel, while leaving other mechanics considerations to future studies, which could include ice crystallization, CPA loading, and CPA toxicity, which are essential for successful vitrification. Finally, this study examines various practical rewarming approaches, including nanowarming and convective heating at the container boundaries.

## 2. Methods and materials

### 2.1. Geometric modeling

**Fig 1:**
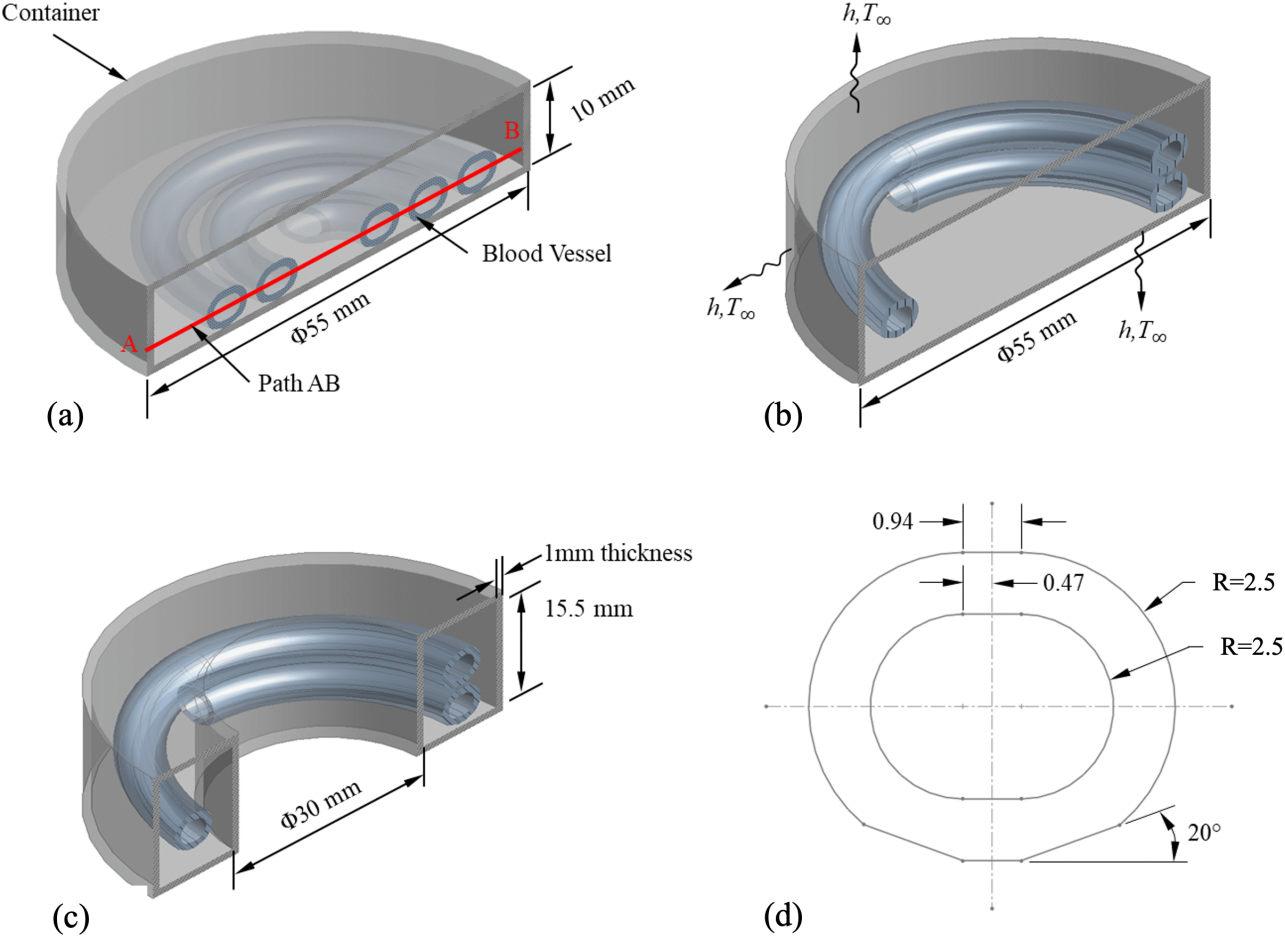
Blood vessel (209 mm in length) and container geometries: (a) a spiral configuration (7 mm pitch, 2.5 revolutions) in a solid cylindrical container filled with CPA up to 8 mm; (b) a helical configuration (24 mm in diameter coil, 1.5 revolutions) in a solid cylindrical container filled with CPA up to 13.5 mm; (c) a helical configuration in a hollow cylindrical container filled with CPA up to 13.5 mm; and (d) a cross section of the blood vessel with internal and external radii of R=1.5 mm and R=2.5 mm, respectively (all dimensions are in mm). Path AB runs across the container at the height of the blood vessel centerline and is used for mechanical stress analysis.

Two basic configurations are considered for the BV: spiral and helical as schematically illustrated in Fig 1. The rationale for the spiral configuration in Fig 1(a) is that it leads to the thinnest sample, potentially leading to a more uniform temperature distribution, which may result in lower thermal stresses. On the other hand, the rationale for the coil configuration in Fig 1(b) is that the BV will experience a uniform temperature distribution even if the temperature across the domain is nonuniform, since the BV is placed at equidistance from the center. To improve temperature uniformity in the latter case while potentially lowering thermal stress, a coiled configuration in a hollow container was also investigated (Fig 1(c)).

In all cases, the geometric model was created with the computer-aided design (CAD) commercial software package SOLIDWORKS 2021. In order to avoid high aspect ratio elements at the interface between the BV, its surrounding CPA, and the bottom of the container, the BV cross section was slightly modified, as illustrated in Fig 1(d). In general, high aspect ratio elements are not well tolerated by the finite elements analysis (FEA) solver and may lead to numerical convergence difficulties, extended computation runtime, and potentially unrealistically high stresses. Contributing to this effect are step-like changes in physical properties between the BV and the bottom of the container, and step-like changes in nanoparticles concentration between the BV and its surroundings. In reality, the diameter, length and thickness of the popliteal artery vary largely based on gender, age, body weight, ethnicity, and race. An external diameter of 6.0 mm for the popliteal artery was selected in this study, as an average value of literature data in the range of 5.4 mm and 6.8 mm [45–47]. The popliteal artery wall thickness was selected to be 1.0 mm, as an average value of MRI measurements on 27 patients [48]. An artery length of 208.7 mm was selected, based measurements from 50 cadavers [49]. Other practical dimensions are illustrated in Fig 1, including the CPA level, details of spiral and coil configurations and cross-section of the blood vessel.

Thermomechanical stress analysis was performed using the commercial FEA software package ANSYS 2020 R2, where the geometry was discretized into tetrahedral elements for thermal and structural analysis using an in-built mesh generator. Based on a mesh convergence study, the spiral package used 90290 elements (SOLID87 and SOLID90) in total for thermal analysis and 64465 elements (SOLID186 and SOLID187) for structural analysis. For the solid-helical configuration totals of 86219 and 88119 elements were used for the thermal and structural analyses, respectively, while the hollow-helical configuration required a total of 155147 and 148402 elements, respectively. A typical run time was between 1–3 hours for thermal analysis and 8–14 hours for structural analysis, using an Intel Core i7-9700 machine (8-core, 12MB cache, 4.7 GHz).

### 2.2. Heat transfer modeling

Consistent with the previous studies [17,23,26,50,51], the heat transfer within the entire domain (CPA and BV) is considered solely by conduction [52], neglecting heat generation due to viscous dissipation and internal convection effects:


$$\begin{array}{c} \rho C_{p}\frac{\partial T}{\partial t}=\nabla \cdot (k\nabla T)+\dot{q} \end{array}$$ (1)

where *ρ* is density, *Cp* is specific heat, *T* is temperature, *t* is time, *k* is thermal conductivity, and $\dot{q}$ is volumetric heat generation during nanowarming. Beyond its very low magnitude, neglecting viscous heat dissipation allows for decoupling of heat transfer and stress problems, which are solved sequentially [17,23,24,26]. Heat transfer within the thin layer of air between the CPA upper surface and the container lid (2 mm) is also assumed to be solely by conduction. This layer is too thin to develop convection cells [52].

The container is cooled by circulating a cold air-nitrogen vapor mixture in the chamber of a commercial controlled-rate cooler [52], which leads to a convective boundary condition on the outer surface of the container:


$$\begin{array}{c} -\hat{n}\cdot (k\nabla T)=U(T_{s}-T_{\infty }) \end{array}$$ (2)

where $\hat{n}$ is a unit vector normal to the outer surface of the container, *U* is an overall heat transfer coefficient, and the subscripts *s* and *∞* refer to the outer surface of the container and air circulated in the cooling chamber, respectively. A practical *U* value of 150 W/m^2^K was selected for this study, being a representative value for an average air velocity of about 25 m/s within the chamber of the controlled rate cooler, such as Kryo 10 (Planar PLC, UK) [53].

### 2.3. Solid mechanics modeling

Consistent with previous studies, a maxwell fluid model is used to simulate the viscoelastic behavior of CPA surroundings and the CPA-loaded BV [12,17,24,40,51,53]:


$$\begin{array}{c} \dot{\varepsilon }={\dot{\varepsilon }}_{elastic}+{\dot{\varepsilon }}_{creep}+{\dot{\varepsilon }}_{thermal} \end{array}$$ (3)

where $\dot{\varepsilon }$ denotes a strain rate tensor, and where the elastic, creep, and thermal strain rates are calculated by:


$$\begin{array}{c} {\dot{\varepsilon }}_{elastic}=\frac{\text{1}}{E}\left[(\text{1}+\nu )\dot{\sigma }-\nu I\cdot tr\left(\dot{\sigma }\right)\right] \end{array}$$ (4)

$$\begin{array}{c} {\dot{\varepsilon }}_{creep}=\frac{\dot{S}}{\text{2}\eta } \end{array}$$ (5)

$$\begin{array}{c} {\dot{\varepsilon }}_{thermal}=\alpha \dot{T}I \end{array}$$ (6)

where *E* is the Young’s modulus, *ν* is the Poisson’s ratio, $\dot{\sigma }$ is the stress-rate tensor, ***I*** is the identity matrix, $\dot{S}$ is the deviatoric stress rate tensor (the difference between $\dot{\sigma }$ and the hydrostatic pressure rate tensor), *η* is the viscosity, *α* is the thermal expansion coefficient, and *tr* is the trace of matrix. Consistent with the prior studies, the CPA is assumed to adhere to the container walls during the cryogenic protocol [19,20,53–55].

### 2.4. Material properties

The reference CPA selected in this study is DP6 + 0.175M Sucrose, which has drawn significant attention in recent years [56]. DP6 is a cocktail of 3M dimethyl sulfoxide (DMSO) and 3M propylene glycol, while the sucrose is added as a synthetic ice modulator (SIM) [41,56,57]. The only literature data on relevant properties of DP6 + 0.175M sucrose are the critical cooling and rewarming rates, being <1°C/min and 15°C/min, respectively [56]. Other thermophysical properties in this study are either approximated as being similar to those of pure DP6 or interpolated from available data on pure DP6 and DP6 + 0.5M sucrose, as described below and listed in Table 1. It is assumed that the thermomechanical behavior of the vitrified specimen is dominated by those of the vitrified CPA, which are taken for the current case studies [24,50,58]. Finally, the container material is modeled as ABS plastic, Table 1.

**Table 1:** Thermal and mechanical properties used for modeling in the current study.

Property	CPA	Acrylonitrile Butadiene Styrene (ABS Container)
Density, kg/m^3^	1064.3 [59]	1070 [60]
Young’s Modulus, MPa	800 [40,61]	2.26 × 10^3^ [62]
Poisson’s Ratio	0.25 [17]	0.38 [62]
Thermal Conductivity, W/m-K	Eq. (7)	0.17 [63]
Specific Heat, J/kg-K	Eq. (8)	1865 [63]
Thermal Expansion Coefficient, K^-1^	Eq. (9)	1.6 × 10^−4^ [62]
Viscosity, Pa ∙ s	Eq. (10)	n/a

Since the thermal conductivity of DP6 + 0.175M sucrose is not available, the thermal conductivity of DP6 is assumed in the current study [57]:


$$\begin{array}{c} k=\text{1}.\text{2}\text{0}\times {\text{1}\text{0}}^{-\text{9}}T^{\text{4}}+\text{4}.\text{1}\text{0}\times {\text{1}\text{0}}^{-\text{7}}T^{\text{3}}+\text{3}.\text{1}\text{1}\times {\text{1}\text{0}}^{-\text{5}}T^{\text{2}}-\text{2}.\text{9}\times {\text{1}\text{0}}^{-\text{3}}T+\text{0}.\text{4}\text{2}\text{9} \end{array}$$ (7)

where the temperature is in Celsius degrees and the thermal conductivity is in W/m-°C.

The specific heat for the DP6 + 0.175M sucrose is interpolated from data on pure DP6 [64] and DP6 + 0.6M sucrose [65,66]:


$$\begin{array}{c} C_{p}=\left\{ \begin{array}{c} @c-\text{0}.\text{0}\text{0}\text{0}\text{5}T^{\text{3}}-\text{0}.\text{0}\text{3}\text{6}T^{\text{2}}+\text{5}.\text{5}\text{9}\text{8}\text{2}T+\text{2}\text{8}\text{6}\text{3}\\ \text{5}.\text{9}\text{7}\text{2}\text{6}T^{\text{2}}+\text{1}\text{5}\text{2}\text{6}.\text{1}T+\text{9}\text{8}\text{2}\text{6}\text{6}\\ \text{7}.\text{8}\text{6}\text{3}\text{1}T+\text{2}\text{0}\text{1}\text{0}.\text{2} \end{array}\right.\;\begin{array}{c} -\text{1}\text{0}{\text{5}}^{\circ }\text{C}<T\\ -\text{1}\text{2}{\text{4}}^{\circ }\text{C}<T\leq -\text{1}\text{0}{\text{5}}^{\circ }\text{C}\\ -\text{1}\text{5}{\text{0}}^{\circ }\text{C}<T\leq -\text{1}\text{2}{\text{4}}^{\circ }\text{C} \end{array} \end{array}$$ (8)

where the specific heat is given in J/kg-°C.

Similar to the specific heat, the thermal expansion coefficient of DP6 + 0.175M sucrose is interpolated from available data on pure DP6 and DP6 + 0.5M sucrose [59]:


$$\begin{array}{c} \alpha =\text{1}.\text{2}\text{5}\text{4}\times {\text{1}\text{0}}^{-\text{6}}T+\text{0}.\text{0}\text{0}\text{0}\text{2}\text{2}\text{8}\text{1} \end{array}$$ (9)

where the temperature is given in Celsius degrees, and the thermal expansion coefficient is given in 1/°C.

Finally, the viscosity of DP6 + 0.175M sucrose received a special treatment, being a parameter dominating the development of thermomechanical stress. Since all vitrifying materials display similar exponential increase of viscosity with the decreasing temperature [67], the viscosity of DP6 + 0.175M sucrose as a function of temperature is assumed similar to that of DP6 but with a temperature shift equal to the glass-transition temperature difference between the two solutions [17]:


$$\begin{array}{c} \eta =\left\{ \begin{array}{c} @c\text{5}.\text{1}\text{1}\times {\text{1}\text{0}}^{\text{4}}\\ \text{5}.\text{6}\text{5}\text{0}\text{3}\times {\text{1}\text{0}}^{-\text{2}\text{3}}e^{-\text{0}.\text{6}\text{2}\text{0}\text{7}T}\\ \text{4}.\text{8}\text{2}\times {\text{1}\text{0}}^{\text{1}\text{4}} \end{array}\right.\;{\begin{array}{c} -\text{1}\text{0}{\text{0}}^{\circ }\text{C}<T\\ -\text{1}\text{3}{\text{7}}^{\circ }\text{C}<T\leq -\text{1}\text{0}\text{0}\\ T\leq -\text{1}\text{3}{\text{7}}^{\circ }\text{C} \end{array}}^{\circ }\text{C} \end{array}$$ (10)

where the viscosity is given in Pa ∙ s, the glass transition temperature of DP6 is −119°C [67] and the glass transition temperature of DP6 + sucrose is −113°C [56]. Note that locking the CPA viscosity at a constant value above −100°C is a choice of practice [17,68], but it does not accurately capture the physics at higher temperatures. In general, selecting a higher value of viscosity in this temperature range reduces computational cost dramatically, with no impact on stress development. The reason being that such a viscosity value is too low for the typical strain rates to give rise to any measurable stress. Furthermore, heat transfer in the CPA in the corresponding temperature range is approximated to be solely by heat conduction, where convective effects have been demonstrated negligible [53].

Following encouraging studies on rat hearts [68,69], this study investigates the possibility of using silica-coated iron oxide nanoparticles (sIONPs) (EMG-308, Ferrotec, Bedford, NH) when excited at an electromagnetic field strength of 62 kA/m and in a frequency of 185 kHz [50,69,70]. The volumetric rewarming rate in that case can be modeled as [23,26,50]:


$$\begin{array}{c} \dot{q}=C_{n}\times SAR;\;SAR=\left\{ \begin{array}{c} @c\text{3}\text{2}\text{3}-\text{4}.\text{6}T\\ \text{6}\text{9}\text{1} \end{array}\right.\;\begin{array}{c} -\text{8}{\text{0}}^{\circ }\text{C}<T\\ T\leq -\text{8}{\text{0}}^{\circ }\text{C} \end{array} \end{array}$$ (11)

where *C*_*n*_ is the concentration of nanoparticle in mg/mL and *SAR* is the specific absorption rate in W/g. Note that the data compiled in Eq. (11) was measured on sIONPs in VS55, but the volumetric heating power is expected to be dominated by the nanoparticles and not by the carrier solution.

### 2.5. Thermal protocol

**Fig 2:**
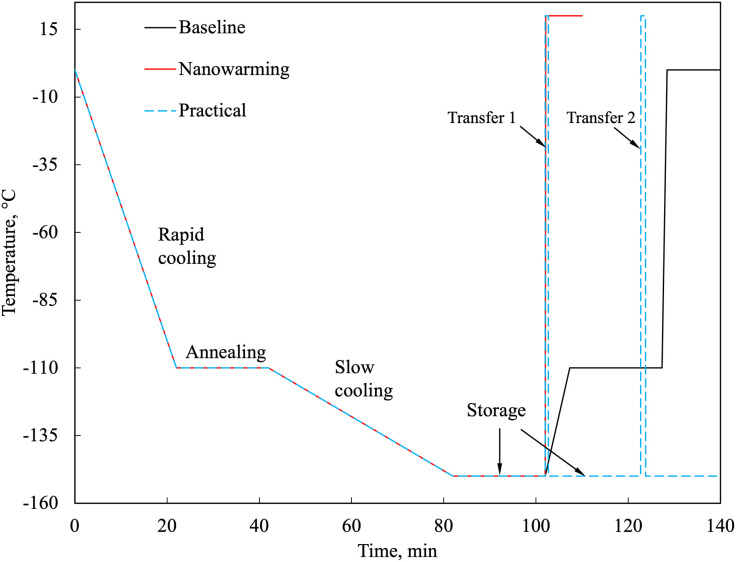
Thermal protocols studied: the curves refer to the thermal history of the convective environment (*T*_*∞*_), the baseline protocol is subject to convective rewarming [17], the nanowarming protocol follows similar phases of cooling and storage but deviate only during rewarming [65], and the practical protocol replicating the baseline protocol but with the addition of thermal exposure to a surrounding temperature of 20°C when the specimen is moved to and from cryogenic storage (annotated as Transfer 1 and Transfer 2, respectively).

Consistent with previous studies [17,23,24], the cooling protocol in this study consists of varying the surroundings temperature (*T*_*∞*_) in four stages (Fig 2), including (i) rapid cooling at 5°C/min from 0°C initial temperature to −110°C, which is 3°C above the glass transition temperature (*T*_*g*_) of DP6 + 0.175M sucrose; (ii) temperature hold for 20 minutes at −110°C, for stress relaxation [17]; (iii) slow cooling at 1°C/min down to storage temperature of −150°C; and (iv) indefinite storage at a temperature of −150°C (practically, the computer simulation continued until the domain reached thermal equilibration). The storage temperature was selected based on the practical temperature range for commercially available coolers below glass transition. While the initial cooling at of 5°C/min ensures that the domain is cooled faster than the critical cooling rate (CCR) of DP6 + 0.175M sucrose (<1°C/min [56]), the slower cooling rate in lower temperatures is deemed a minimum practical rate in a controlled-rate cooler, which is aimed at reducing thermal stress [23,26,39]. The stress relaxation stage at −110°C (or annealing stage), is selected to minimize residual stress [17].

Being the focus of this study, two practical rewarming scenarios are considered: rewarming by convection on all external surfaces of the domain, and volumetric heating using nanoparticles. The first practical rewarming scenario is for convective heating with no nanowarming, consisting of four stages, where warming rates refer to the rate of temperature change in the convective surrounding: (i) slow warming from storage temperature of −150°C to −110°C at a rate of 7.5°C/min; (ii) temperature hold at −110°C for 20 minutes, which is sufficient for the specific domain to approach thermal equilibrium; (iii) rapid rewarming at the rate of 100°C/min up to 0°C; and (iv) temperature hold at 0°C thereafter. The overall heat transfer coefficient during surface rewarming is 150 W/m^2^K during both cooling and rewarming, which is characteristic to a controlled-rate cooler [13,20,51].

The second practical rewarming scenario is for nanowarming, which may be combined by various intensities of convection on the boundary: none (mathematical insulation), free convection (25 W/m^2^K), or forced convection (150 W/m^2^K) as in the first scenario in the absence of nanowarming. In either case on nanowarming, recovery from cryogenic storage was achieved by a single step from cryogenic storage to room temperature of 20°C (i.e., no annealing during rewarming) [39,50,68,70]. While nanowarming has the potential of heating the specimen uniformly, if dispersed evenly in the domain, practical limitations on CPA perfusion and nanoparticles loading may affect this ideal effect [50]. This would result in lower heat generation inside the lumen compared with the surrounding tissue, depending on the achievable nanoparticle loading. This study considers various plausible combinations of nanoparticle concentrations within the lumen and in the surrounding media, as listed in Table 2. Based on a preliminary computational analysis in this study, a minimum nanoparticle concentration of 10 mg-Fe/mL was selected for the surrounding CPA in order to outperform purely convective heating. The investigated cases listed in Table 2 represent various combinations of volumetric and boundary heating.

**Table 2:** Summary of nanowarming case studies: nanoparticles concentration, boundary conditions, and the resulting maximum major principal stress.

Case	Nanoparticle Concentration, mg-Fe/mL		Boundary Condition*	Maximum Major Principal Stress, MPa	
	Inside BV	Around BV		BV	CPA
I	10	10	Forced convection	2.55	6.69
II	10	15	Forced convection	2.78	4.71
III	15	10	Forced convection	2.39	5.24
IV	5	15	Forced convection	3.11	2.51
III(a)	15	10	Insulation	0.53	1.30
III(b)	15	10	Natural convection	0.21	1.00

* Selected convective heat transfer coefficient for forced convection, natural convection, and insulated boundary condition are 150 W/m^2^-K, 25 W/m^2^-K, and 0 W/m^2^-K, respectively.

A practical case is also examined (Fig 2), where the surrounding temperature fluctuates due to specimen handling. This case was observed in the lab, when a cooled specimen is transferred from the chamber of a controlled-rate cooler to a storage refrigerator and back, while being exposed to ambient temperature for a brief period (1 minute in this example).

The above thermal protocols resulted in a cooling rate range at the slowest responding point in the domain as follows: 2.6°C/min to 5.3°C/min for the spiral cases, 1.8°C/min to 5.6°C/min for the helical cases, and 3.7°C/min to 5.5°C/min for the hollow helical cases. With a CCR of <1°C/min, these thermal protocols ensure vitrification for the CPA under consideration. The convective rewarming cases resulted in a rewarming rate range at the slowest responding point in the domain as follows: 5.6°C/min to 21.0°C/min for the spiral cases, 3.3°C/min to 10.3°C/min for the helical cases, and 7.7°C/min to 27.3°C/min for the hollow helical cases. While the CWR for the specific CPA is 15°C/min [56], which suggests rewarming-phase crystallization (RPC) [71] in some cases, they served for reference only, and expending the thermomechanics analysis to include crystallization was deemed unwarranted. In contrast, the application of nanoparticles—the focus of the current study—resulted in two orders of magnitude faster rewarming rates which suggests RPC avoidance. For instance, the rewarming rates at the corresponding locations for the natural convection boundary condition ranged from 73.2°C/min to 146.4°C/min. Note that RPC is an all-inclusive term to describe ice nucleation (devitrification) and growth (recrystallization) during rewarming [23,56,65].

## 3. Results and discussion

A comprehensive approach to reduce thermal stress would call for an optimization process involving numerous parameters pertaining to the container geometry, cooling protocol, rewarming method, CPA composition, and CPA loading and unloading, to name a few. While such an ambitious goal is beyond the scope of the current study, this study focuses on selected effects and conditions that are deemed important and closely related to ongoing preservation studies [25,32,68].

First, the criterion indicating the likelihood of structural damage in glassy materials is presented in brief for the completeness of presentation [12,72]. For this purpose, it is most convenient to imagine a stationary cubic unit volume of a vitrified material, and account for all the forces acting on its surfaces. The mechanical stress in this small unit volume is conventionally described as a nine-component tensor, three representing normal stresses and six representing shear stresses. Three pairs of normal forces are acting on the opposing cube faces, each pair of equal forces but in opposing directions, which result in three normal stresses. Similarly, two equal in magnitude but opposing in direction pairs of shear forces act on every two opposing faces of the cube, resulting in a total of six shear stress.

While the stress in the material is independent of the coordinate system used to describe the above unit volume, the relative magnitudes of the above nine components of the stress tensor do depend on the orientation of that system. Furthermore, it is possible to rotate that coordinate system such that all the shear stresses vanish, a case in which one of the normal stresses reaches a maximum—that is the *major principal stress*, which is also known as the *first principal* stress. At this control volume orientation, ordering the normal stresses from higher to lower values results in the second and third principal stresses, where the latter is also known as the *minor* principal stress. While the major principal stress is algebraically the highest normal stress at a point, which can be positive (tensile) or negative (compressive), it is often tensile in common scenarios relating to cryopreservation by vitrification [12,14,17,23,24,51,55,71]. While the first (major), second, and third (minor) principal stresses are conventionally annotated *P*_1_, *P*_2_, and *P*_3_, the analysis in this focuses only on the first principal stress, as it only seeks to identify the onset of fracturing, and the first principal stress is simply annotated as *P* with a subscript referring to specific configurations.

Vitrified (glassy) CPAs behave as brittle materials, which are characterized by high strength in compression and low strength in tension [12,14,72]. For this reason and for the simplicity in the current discussion, the vitrified material is considered at risk of structural failure when the major principal stress exceeds the strength of the material in tension [17,54]. While the tensile strength of vitrified CPAs is only sparsely available [19], 3.2 MPa serves as a good representative strength-to-fracture in vitrified 7.05M DMSO [19]. More broadly, given the fact that fracturing in a brittle material is a stochastic event, tensile stress levels in the order of MPa are generally considered hazardous herein. Notably, structural failure in brittle materials is highly susceptible to preexisting material flaws, and especially in areas of stress concentration (i.e., local stress much higher than the average) [51,53].

### 3.1. Packaging configuration

**Fig 3:**
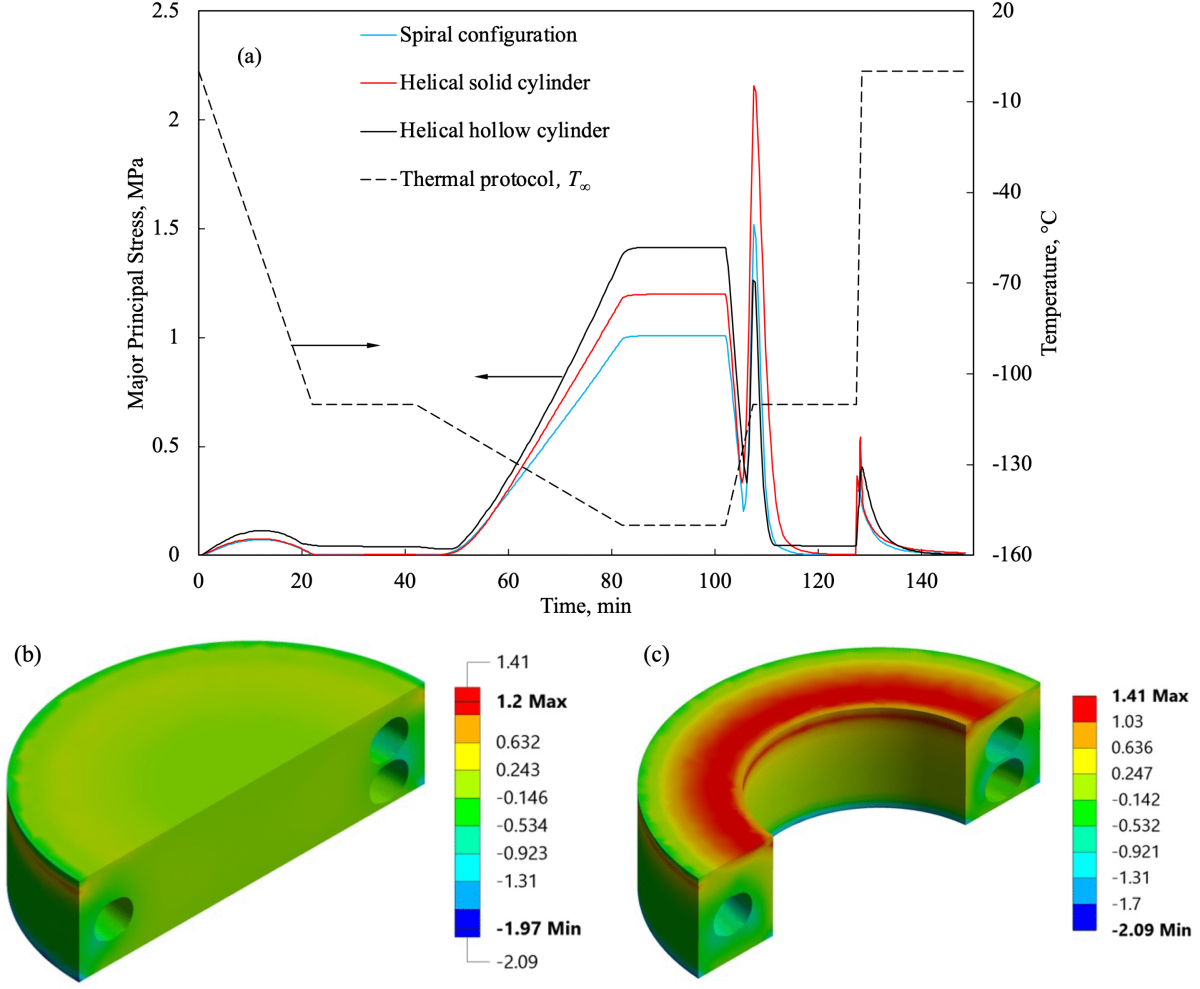
Major principal stress in the surrounding CPA for the baseline thermal protocol (Fig 2): (a) maximum major principal stress history for the three configurations illustrated in Fig 1; (b) major principal stress distribution overlayed on a cross-section illustration of the solid-helical configuration during cryogenic storage; and (c) major principal stress distribution overlayed on a cross-section illustration of the hollow-helical configuration during cryogenic storage. The BV and container are omitted in the cross-section illustrations to aid visualization of stress in the CPA.

Previous studies investigated thermal and thermomechanical effects of various container configurations for specific applications and organs, such as the heart [68], the kidney [54,58], and the ovary [73]. Another study demonstrated scaling effects for the cryobag, as a special-case container [24]. One of the objectives in the current study is to demonstrate how a container shape can be tailored to the unique case blood vessels cryopreservation by vitrification. Fig 3 displays the major principal stress (*P*) for the three configurations studied, where positive values indicate tension and negative values compression. The corresponding locations for the *P* curves in Fig 3(a) may vary during the thermal protocol based on a host of conditions but it is not known *a priori*. To provide some insight, Fig 3(b)-3(c) display the *P* distribution during cryogenic storage for the two helical cases.

The *P* values in Fig 3(a) are low above the selected annealing temperature of −110°C (i.e., 3°C above *T*_*g*_), when the CPA viscosity is relatively low and the material is free to flow easily. In general, stress relaxation time is directly proportional to the viscosity, which increases exponentially with the decreasing temperature. In practice, the specific relationship between relaxation time and temperature is complex, but the application of a temperature-hold a few Centigrade degrees above *T*_*g*_ to minimize residual stresses as the material transitions to glass is well established [17,74]. The term residual stress is used to signify stress locked into the material due to glass transition, which do not dissipate as the vitrified material reaches thermal equilibrium during cryogenic storage.

Notably, the thermal protocol curve presented in Fig 3 is of the surrounding temperature (*T*_∞_), while the thermal response within the domain lags behind due to the underlying principles of heat transfer. For this reason, the development of significant mechanical stress also lags behind, with the example of increasing *P* in some time delay after the onset of the second cooling portion of the thermal protocol past annealing. The maximum *P* during cooling appears in tension as the domain approaches thermal equilibrium at the storage temperature (essentially a residual stress), where *P*_*spiral*_ = 1.0 MPa, *P*_*solid*_ = 1.2 MPa, *P*_*hollow*_ = 1.4 MPa, Fig 3(a). Counterintuitively, the hollow helical configuration yielded the highest stress during cooling, but *P* reached a hazardous level in all cases.

Initial rewarming from cryogenic storage but below *T*_*g*_ leads to decrease in thermal stress, but the intermediate hold temperature during rewarming at −110°C brings a quick spike of stress. The onset of continued rewarming from cryogenic storage is associated with a sudden increase in stresses where the specimen is most prone to fracturing, which is consistent with previous theoretical [17,23,54,75,76] and experimental [31] studies. Clearly, the hold temperature at 3°C above *T*_*g*_ stops this increase in stress and facilitates stress relaxation. Finally, a second increase in stress is observed at the onset of rapid warming past the annealing stage, but it dissipates quickly as the viscosity of the CPA decreases exponentially with the increasing temperature.

Although the hollow helical configuration yielded the highest stresses during cooling and cryogenic storage, it also yielded the lowest stresses during rewarming, among the three configurations tested, where *P*_*spiral*_ = 1.5 MPa, *P*_*solid*_ = 2.3 MPa, *P*_*hollow*_ = 1.25 MPa. In fact, the hollow helical configuration is the only one to yield similar stresses during cooling and rewarming, which is considered the most favorable outcome. However, this outcome is not only dependent on the configuration but also on the design thermal protocol [17]. It follows that the thermal protocol and the geometric setup need to be optimized together. Clearly, all maximum stresses during rewarming are also significant and potentially harmful to structural integrity.

Refocusing on Figs 3(b) and 3(c), the location of the maximum stress is found furthest from the center in the solid-helical case, but on the edge of the inner surface in the hollow-helical configuration. The history and distribution of stress is also related to the total volume contained in the system, which is 14.9 mL, 26.9 mL, and 17.4 mL for the spiral, solid-helical, and hollow-helical configurations, respectively. While the three different packaging configurations were simulated to follow the same practical thermal protocol [17,24], it is conceivable to further tailor the thermal protocol to the specific geometries and thereby reduce stresses.

**Fig 4:**
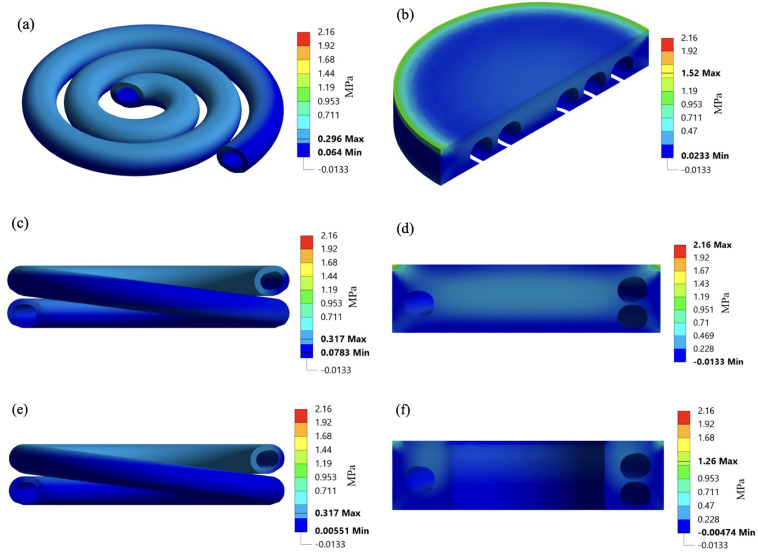
Major principal stress distribution overlayed on the blood vessel and a cross section of the CPA: (a)-(b) spiral geometry, (c)-(d) solid cylindrical spiral geometry, and (e)-(f) hollow cylinder helical geometry, respectively, as illustrated in Fig 1. Panels (a), (c), and (e) refer to the BV wall, whereas (b), (d), and (f) refer to the surrounding CPA. All results correspond to the time at which the major principal stress reaches a maximum in the domain during rewarming part of the baseline protocol.

Fig 4 displays the *P* distribution separately in the blood vessels and the surrounding CPA at the beginning of annealing stage during rewarming subject to convective boundary conditions. It is a bit difficult to draw general conclusions when comparing the stress levels between cooling and rewarming, since the rate of temperature changes (i.e., cooling rate and warming rate) varies over time and between cases, and so is the strain rate. In general, the strength-to-fracture decreases with the increasing strain rate, and the strain rates observed in this study are thought to be low to moderate.

As can be seen from Fig 4, the stress level in the blood vessel in all configurations is comparable, while the maximum stress in the entire domain varies significantly. Notably, fracture formation is expected once the stress exceeds the strength of the material at any location. However, once it is initiated in a brittle material, its propagation can continue under much smaller stresses and at extremely high rates [77]. While modeling fracture dynamics is quite complex and well beyond the scope of this study, it follows that one should be more concerned about the maximum stress, even if not found within the blood vessel region.

It is noted that the CPA-air interface deforms during cooling while the mechanical stress builds up [20,55]. This effect may become significant in tall and narrow containers, which can lead to high stress concentrations and fracture [51,53,55]. Similar surface deformations in the current study are less than 0.5 mm in all cases studied. This low deformation value is due to the low height-to-width ratio of the container, where the actual deformation cannot be easily observed from the presented figures. In general, the FEA becomes more complex when large deformations are at play [20,51,55,71].

### 3.2. Nanowarming

Given relevancy to ongoing studies [8,39,50,69,70,78], this study focuses on iron oxide nanoparticles (IONPs) mixed in the CPA, while following the underlying assumption that nanoparticles remain in the organ vasculature space or the surrounding CPA, but not in the BV walls. For this heating system to work, the vitrified specimen is placed in a radiofrequency (RF) electromagnetic field, while heat is being generated in the nanoparticles as response to the alternating magnetic field [39]. In the case of complex organs such as heart [50,68,69] or kidney [54,58,70], nanoparticle loading is governed by flow limitations through the vasculature. Suboptimal nanoparticles distribution adversely affects heating uniformity, which may elevate thermal stresses [50,68]. Fortunately, the distribution of nanoparticles can be better controlled during the preservation of a single BV, as is the case in the current study.

With improved nanoparticles loading, one may envision loading the BV lumen with a different concentration of nanoparticles for improved thermal control. While opposing arguments can be made for both increased and decreased concentration of nanoparticles within the BV lumen relatively to the surrounding CPA, this study examines both scenarios, with representative cases listed in Table 2. The spiral BV configuration was selected for the purpose of this case study, which demonstrated the lowest residual stress for the above investigated protocol (Fig 3). The nanowarming analysis included three possible boundary conditions: thermal insulation, free convection, and forced convection, with the latter condition already also investigated early in this study (Table 2). The selection of nanoparticle concentrations and boundary conditions is not a result of a systematic optimization process, but rather an exploratory selection to identify preferable trends.

The rationale behind selecting the five cases in Table 2 is as follows. Case I is the base case studied above in the absence of nanoparticles and now investigated in an IONPs concentration of 10 mg-Fe/mL, consistent with previous studies [8,50,68,69]. Cases II and III are similar to Case I with the exception that Case II has a 50% higher IONPs concentration in surrounding CPA, while Case III has a 50% higher IONPs concentration in CPA contained in the lumen. Based on results from the Cases II and III, Case IV combines 50% higher IONPs concentration in surrounding CPA and a 50% lower IONPs concentration in CPA contained in the lumen. Based on the results from Case III, Cases III(a) and III(b) explore the scenarios of mathematical insulation (rewarming is achieved solely by internal heat generation) and reduced convective heat transfer (free instead of forced convection).

**Fig 5:**
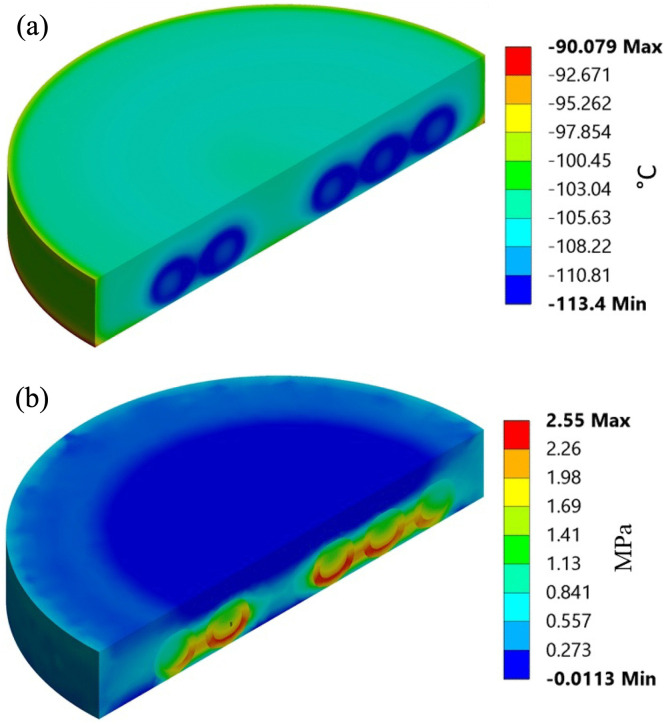
Combined nanowarming and forced convection in Case I (Table 2): (a) temperature field and (b) major principal stress distribution, at the instant in which maximum major principal stress is observed in the blood vessel.

Fig 5 displays the temperature field and the resulting *P*_1_ distribution in reference Case I, at the point when *P* reaches maximum value in the BV during rewarming. Here, although the IONPs concentration in the lumen and the surrounding temperature is identical, the BV walls are colder by about 23°C from the warmest point in the surrounding CPA. This is a significant temperature difference, especially when the wall thickness is only 1 mm (recall no IONPs in the BV wall). At this instant *P*_*max*_ = 2.55 MPa (within the blood vessel wall), in comparison with a value of 1.5 MPa in the reference case of no IONPs. It follows that while heat is now being transferred not only from the surrounding but also generated within the domain, and thereby allow for higher rewarming rates, it adversely affects the resulting stresses, making the specimen more prone to fracturing during rewarming.

**Fig 6:**
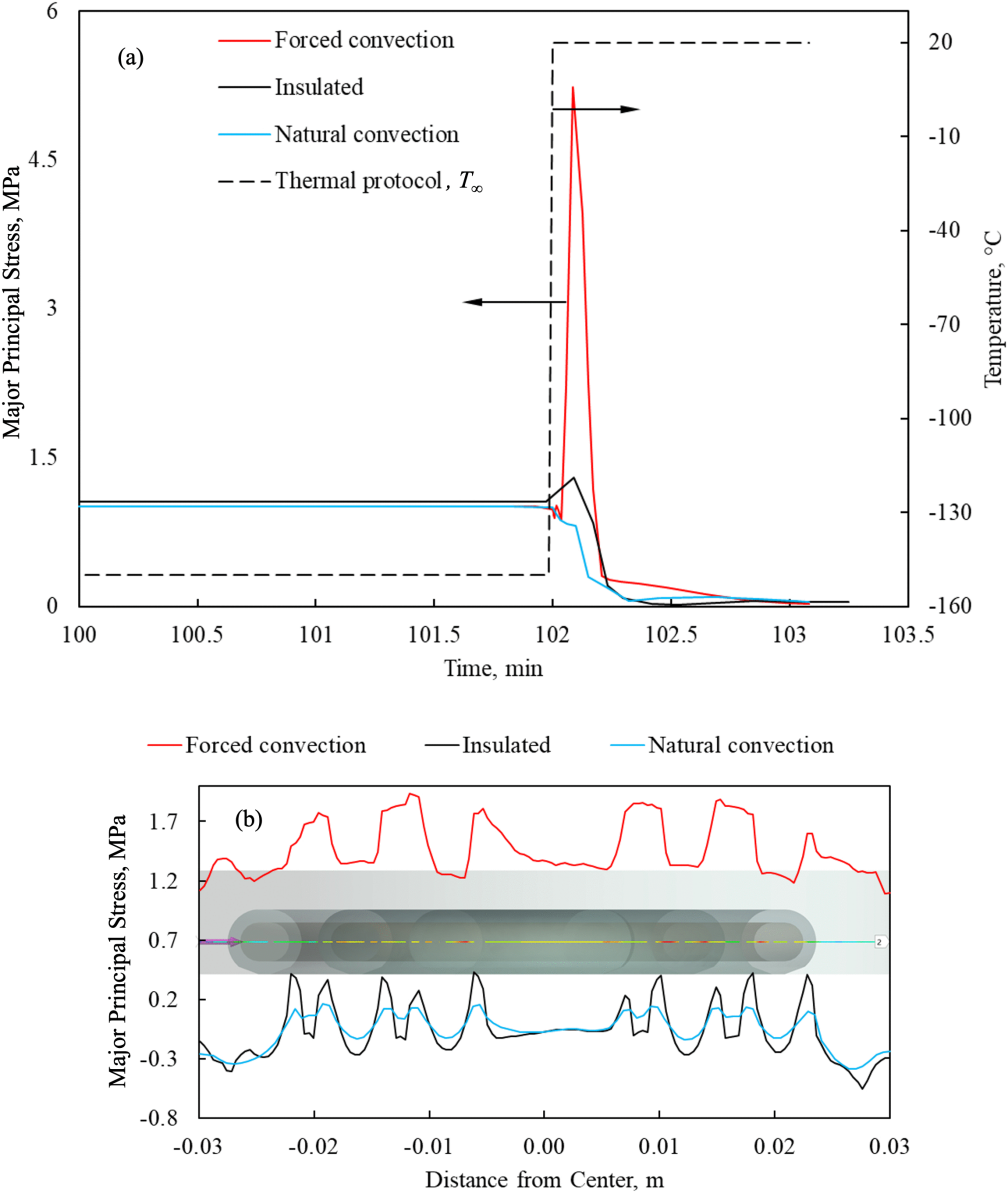
Major principal stress for the spiral configuration during nanowarming: Comparison of boundary conditions of forced convection (Case III), insulation (Case III(a)), and free convection (Case III(b)) (Table 2): (a) temperature and maximum major principal stress histories, and (b) major principal stress distribution along path AB in Fig 1 when the stress is maximum in the domain during nanowarming.

Notably, the maximum stress during nanowarming with a forced convection boundary condition (Cases I-IV) is observed shortly after the onset of rapid rewarming, as illustrated in Fig 6(a) for Case III as an example. Case III is associated with the lowest *P*_1_ out of those four cases. When comparing these cases, the difference in peak stresses is associated with the variation in nanoparticles concentration between the lumen and the surrounding medium, which suggests that this ratio is a parameter to be considered during process optimization. However, the analysis of all the four cases discussed here suggests maximum major principal stress in the order of MPa, which is considered hazardous for preserving structural integrity.

While the maximum stress in the BV was highlighted thus far, it is essential to evaluate the stress in the entire domain since the strength to initial fracture in glassy materials is much higher than the strength at the fracture front, where both the strain rate and stress concentration are very high. Table 2 lists the maximum stress in the domain which is even higher in the first three cases than that in the BV wall. These values only increase the risk of structural damage.

In effort to identify lower-stress protocols, two additional cases of free convection (Case III(b)) and mathematical insulation (Case III(a)) were considered, where mathematical insulation is the limiting case for practical thermal insulation. These two additional cases are imposed on the nanoparticles distribution tested in Case III (15 mg-Fe/mL in the lumen and 10 mg-Fe/mL in the BV), which yielded the lowest *P*_1_ among the first four cases. These additional cases are also considering the spiral BV configuration for reference.

Fig 6(a) displays the maximum *P*_1_ in the domain, whether it is in the BV or surrounding medium, during rewarming phase for cases III, III(a) and III(b). Notably, the intermediate Case of free convection, Case III(b), yielded the lowest overall stresses, where the stress spike during rewarming was completely eliminated. In fact, the maximum *P*_1_ only declined during rewarming in that case from its cryogenic storage value (i.e., from its residual stress level). It is significant and might be counterintuitive that this behavior is observed in the case of natural convention and not in the limiting case of mathematical insulation, which highlights the complexity of the process.

The reason for that counterintuitive observation can be explained as follows. While uniform-distributed heat sources (i.e., nanoparticles) within an insulated boundary condition would result in uniform warming rate (and hence the intuition), the case in point includes container walls which are not internally heated. In order to compensate for that, the parametric study herein also includes convective heating on the outer surface of the container wall. It appears that forced convection leads to overcompensation while free convection is superior. The optimal convective heating rate may change as the distribution of nanoparticles varies between the BV and its surroundings.

**Fig 7:**
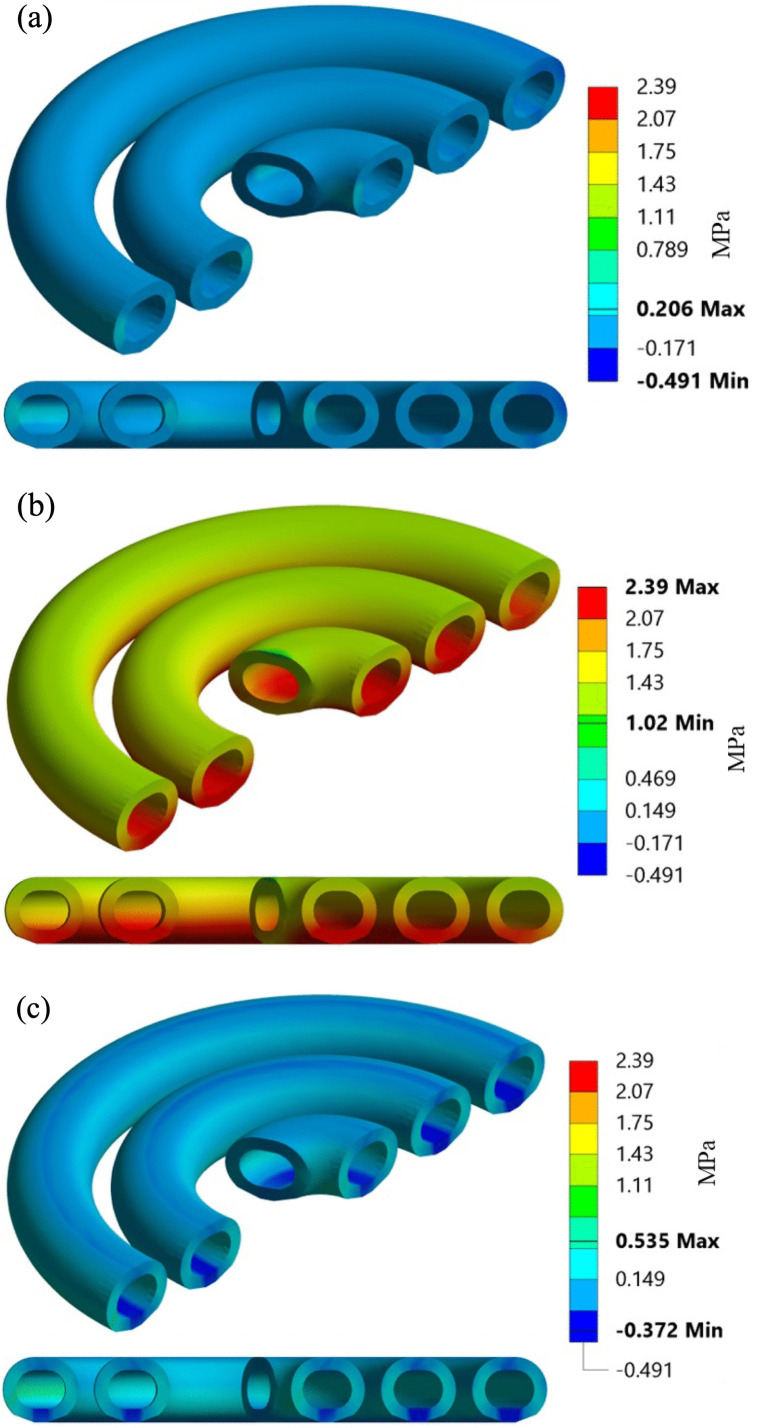
Major principal stress distribution overlayed on the blood vessel surfaces for various boundary conditions: (a) forced convection (Case III), (b) insulation (Case III(a)), and free convection (Cases III(b)) when the stress in the BV reaches maximum during nanowarming (Table 2). The stress scale is common to the three cases, while the maximum and minimum stress level is highlighted with bold numbers for each individual case.

Fig 6(b) displays stress distribution across the diameter of the container, at the height of the centerline of the BV (path AB in Fig 1), in Cases III, III(a), and III(b), at the instant in which *P*_1_ in the domain is maximum. From these cases, forced convection yields the highest principal stress distribution (Case III), while the other two cases yielded fourfold lower stresses. Furthermore, Case III always resulted in tensile stress, while stresses in the other two cases ranged periodically from compressive or tensile. For further detail, Fig 7 displays the *P*_1_ distribution superimposed on the blood vessel surfaces.

While the discussion here focuses on the effects of nanowarming and boundary conditions on the resulting thermomechanical stress, it is reminded that the primary benefit of nanowarming was initially perceived to be as a measure to increase the overall rewarming rate and, thereby, outrunning rewarming phase crystallization (RPC) [23,26,56,65]. Evidently, nanowarming accelerates heating where the average rewarming rate between −113°C and −39°C (*T*_*g*_ and melting point for the case of DP6 + 0.175M sucrose, respectively) in Case III is 155.2°C/min, while in the absence of nanoparticles (i.e., the baseline case) is 3.5°C/min. Since the critical cooling rate of DP6 + 0.175M sucrose to prevent RPC as an example is 15°C/min [56], nanowarming delivers the desired improvement in heating. However, the addition of nanoparticles cannot be generalized as a stress decreasing measure. Instead, it must be qualified for specific geometries, boundary conditions, container material, and nanowarming parameters (type, dispersion, excitation, etc.). It follows that the cryogenic protocol must be optimized for the multiphysics effects associated with reducing mechanical stress and heating rates simultaneously, where the desired outcome from the different physics modes might pose competing needs, based on the special case.

### 3.3. Practical thermal considerations during specimen handling

**Fig 8:**
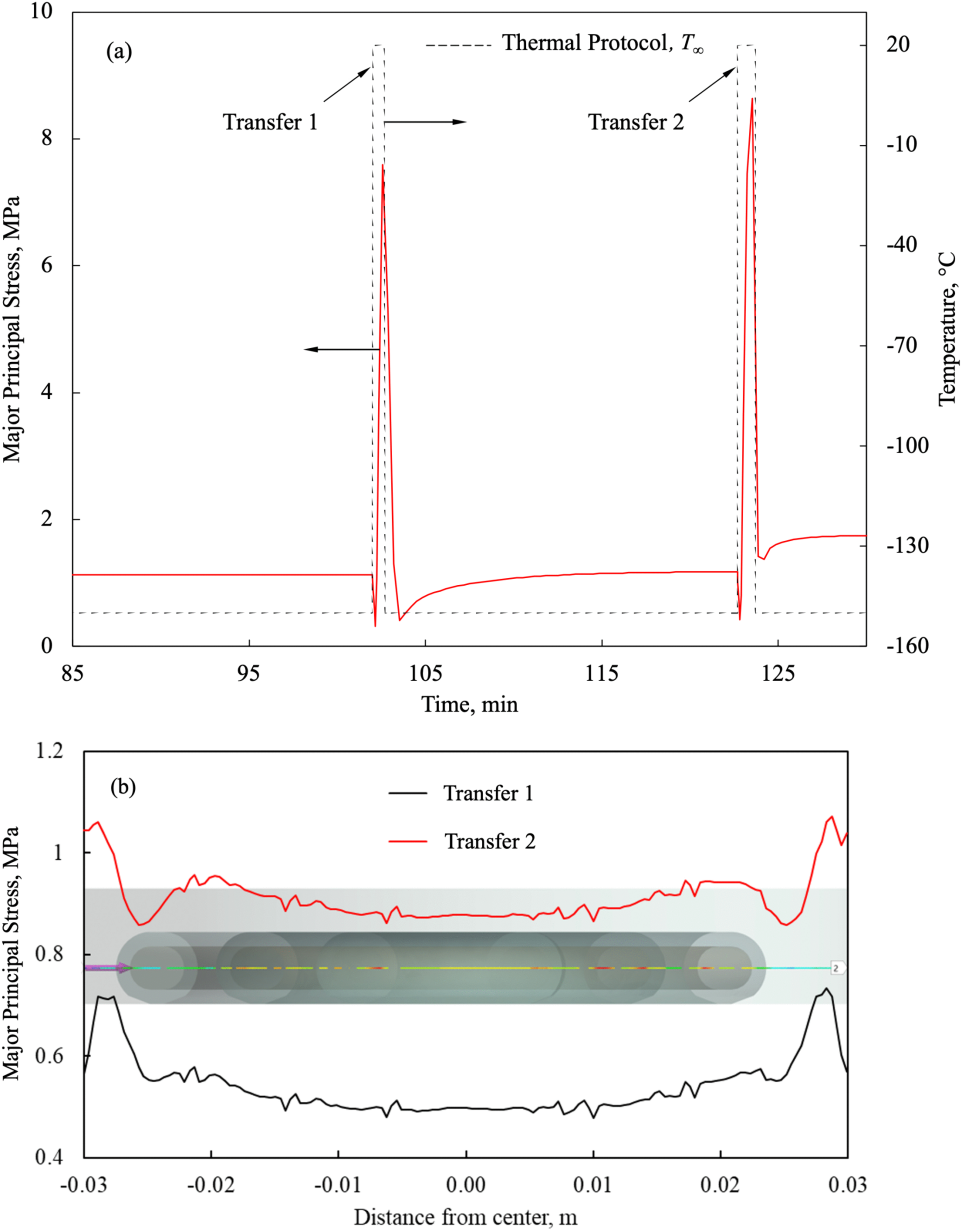
Mechanical stress resulting from thermal exposure to the surroundings during specimen transfer: Transfer to and from storage are noted as Transfers 1 and 2, respectively: (a) maximum major principal stress history, and (b) major principal stress distribution along path AB in Fig 1 at the end of each transfer.

Thus far the discussion addressed idealized cases without considering the need to transfer the specimen between the cooling system (e.g., controlled-rate cooler), cryogenic storage (e.g., refrigerator), and the rewarming apparatus (e.g., controlled-rate cooler, RF heating setup, or a combination of both). In practice, the specimen might be exposed to room temperature as it is transferred to and from cryogenic storage, which may lead to rapid temperature fluctuations and consequently result in a thermomechanical impact. In order to investigate a realistic scenario as an example, we measured the average specimen transfer time during blood vessels experiments by an experienced technician in related studies [70,78], and found them to be 40 s for transfer to cryogenic storage, and 1 min for transfer and setup in a nanowarming apparatus. During transfer, the specimen container, initially at −150°C, is assumed to be exposed to the surroundings at 20°C and free convection (i.e., 25 W/m^2^K). These exposure times are displayed on Fig 2, while the resulting *P*_1_ is displayed on Fig 8(a).

It can be seen from Fig 8(a) that the maximum *P*_1_ in the domain during the first and second transfers are 7.6 MPa and 8.6 MPa, respectively, which are rising from a residual stress of 0.8 MPa. Respectively, the maximum temperature at the container surface rises by only 62.8°C and 101.9°C during the first and second transfers. Fig 8(b) displays the *P*_1_ distribution across the container and blood vessel (path AB in Fig 1(a)) at the time in which the maximum *P*_1_ is observed in the domain. Clearly, *P*_1_ distribution across the domain in Fig 8(b) is lower than the maximum value in the domain by almost and order of magnitude. Interestingly, *P*_1_ values in Fig 8(b) during the first transfer are lower than the residual stress value before and after the transfer. While these stress levels are perceived as somewhat lower than the strength to fracture of the vitrified material, facture will initiate at the point of maximum stress and propagate from there. As discussed above, the resistance of the material to a rapidly progressing facture is much lower than the resistance to its onset due to localized stress concentration and rapid strain rates in the vicinity of the facture edge. While the current analysis represents only one special case of thermal exposure to the surroundings, the resulting impact on structural integrity in the vitrified material must be considered carefully when designing a practical lab protocol.

## 4. Summary and conclusion

The development of thermomechanical stress during cryopreservation by vitrification of the popliteal artery is investigated in this study. The long-term objective in this line of research is to lower thermomechanical stress and thereby reduce the risk of structural damage. The specific objective in the current study is to explore packaging configurations and protocols which may reduce those stresses and can provide directions for cryopreservation optimization. Unlike the case of large organs cryopreservation, the popliteal artery can be packed in configurations and geometries that help reduce adverse thermomechanical effects. This study explores two basic configurations, namely a spiral and a helical blood vessel arrangement in cylindrical containers. Additionally, this study explores the advantages of a hollow cylindrical container, due to its extended surfaces to heat convection and reduced thermal mass.

With the vitrified sample behaving as a brittle material, which is more prone to structural damage under tension, this study focuses on the major principal stress as a key indicator of the likelihood of fracturing. Specifically, the analysis seeks to identify the time and location at which the major principal stress history reaches maximum, which are unknown *a priori*. This study includes ideal scenarios of convective rewarming, nanowarming, and a combination of both.

Results of this study indicate that the maximum stress in the BV is comparable in all three configurations tested and may get to hazardous levels. The maximum stress in the CPA in these cases is much higher than in the BV and might surpass the strength to fracture of the vitrified CPA. Due to the underlying principles of fracture mechanics, once a fracture is initiated anywhere in the domain it can rapidly propagate across it, and even through areas where the average stresses are lower than the onset stress to fracture. The helical BV configuration in a hollow cylinder resulted in the highest stress during cooling out of the cases studied, but the lowest during rewarming.

This study demonstrates that the thermomechanical stress during BV cryopreservation can be lowered below hazardous levels by careful selection of nanoparticle concentrations in the BV lumen and surrounding CPA, in combination with a matching convective boundary condition. This study demonstrates how this combination can be tailored to various configurations. However, this study does not present any optimum thermomechanical stress scenario, which is left for future studies, to be tailored to the specific properties and kinetics of crystallization of CPAs of interest.

Finally, a special case is considered to explore the effects of transferring the specimen between cooling instrumentation during cryogenic storage. This study demonstrates that even a brief exposure to ambient temperature of 1 min or less can cause sudden stress spikes in the specimen, potentially exceeding the strength of the material. This special case demonstrates the need to carefully consider the feasibility and practicality of lab protocols when aiming to reduce the likelihood of structural damage.
